# More severe toxicity of genetic polymorphisms on MTHFR activity in osteosarcoma patients treated with high-dose methotrexate

**DOI:** 10.18632/oncotarget.23222

**Published:** 2017-12-14

**Authors:** Lu Xie, Wei Guo, Yi Yang, Tao Ji, Jie Xu

**Affiliations:** ^1^ Musculoskeletal Tumor Center, Peking University People’s Hospital, Beijing 100044, China

**Keywords:** osteosarcoma, MTHFR polymorphism, drug toxicity, survival

## Abstract

5,10-Methylenetrahydrofolate reductase (MTHFR), a key enzyme for folate metabolism, catalyses the irreversible conversion of 5,10-methylenetetrahydrofolate to 5-methyltetrahydrofolate, which is located at the end of the short arm (1p36.3). Two common non-synonymous variants, the C677T (Ala222Val) and A1298C (Glu429Ala), were mainly described with decreased enzymatic activity and an alteration of intracellular folate distribution. Osteosarcomas are currently treated with high dose of methotrexate (MTX). The decreased enzyme activity of MTHFR theoretically could increase the drug action of MTX and at the same time increase toxic and side effect. Germline variants of C677T and A1298C were studied in 59 osteosarcoma patients, with whom the A1298C is detected with particularly low rate of mutant genotype (*N* = 1, 0.8%) and could not proceed with statistical calculations. 15 patients were wild type of C677T (CC, 25.4%), 20 were heterozygous mutant genotype (CT, 33.9%) and 24 were homozygous mutant genotype (TT, 40.7%). Patients harboring the TT/CT genotype had the same progression-free survival and tumor necrosis rate in comparison with patients having the CC genotype (*P = 0.349 and P = 0.465* respectively). And the C677T polymorphisms had no significant correlation with MTX initial plasma concentration (*P = 0.867; r = 0.024*) and delayed elimination (*P = 0.305; r = −0.136*). However patients with mutant genotype of C677T were associated with higher degree of liver toxicity (*P* = 0.043) and fever reaction of MTX (*P = 0.050*) while G3/G4 hematologic toxicity were more likely to be noticed with TT than CT/CC (*P = 0.095*). The study suggests that genetic polymorphism of MTHFR C677T in the MTX metabolic pathway seems to be associated with the trend for more side effects statistically, but has no obvious effect on histologic response and survival.

## INTRODUCTION

**Figure 1:**
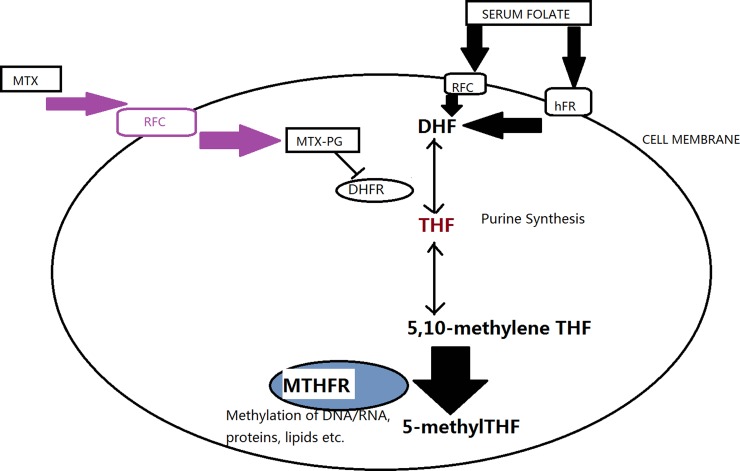
A simplified view of the folate metabolism pathway and the main targets of MTHFR (Transporters: hFR, human folate receptor; RFC, reduced folate carrier. MTX, methotrexate; MTX-PG, methotrexate-polyglutamated; DHF, dihydrofolate; DHFR, dihydrofolate reductase; THF, tetrahydrofolate; MTHFR, 5,10-methylenetetrahydrofolate reductase).

Pharmacogenetics represents a promising future for the individualization of therapy. Several genetic polymorphisms and haplotypes have been investigated in an attempt to optimize therapy with specific drugs. However up to now their clinical applications have still been controversial [1]. 5,10-Methylenetrahydrofolate reductase (MTHFR), which has been described as being located at the branch point in directing folate metabolites toward remethylation of homocysteine and restraint of DNA and RNA bio-synthesis [2], catalyses the irreversible conversion of 5,10-methylenetetrahydrofolate to 5-methyltetrahydrofolate (Figure 1). This gene consists of two well-described polymorphisms: C677T and A1298C [3, 4]. It is reported that the MTHFR C677T genotype decreases by 30 percent of the MTHFR enzyme activity *in vitro* compared with the wild type [5]. And the A1298C causes conformational changes within the MTHFR enzyme that alters the activity of the enzyme but with a lower degree compared to C677T [4].

A large number of published studies have investigated the potential role of MTHFR polymorphisms on toxicity and response to methotrexate(MTX)-based cancer chemotherapy and anti-inflammatory therapy [6–11]. The decreased enzyme activity of MTHFR theoretically could increase both the drug action of MTX but also the toxic and side effect [12]. At the same time MTHFR is also involved in MTX activity by modulating the intracellular pool of folate [13]. Thus theoretically the homozygous or heterozygous mutation of MTHFR should be associated with more therapeutic and toxic effect of MTX-based chemotherapy for malignancies. However from past literatures [12, 14–16], the clinical observation results differed obviously, some of which indeed supported better drug-related prognosis or toxicity while others had negative results.

**Figure 2:**
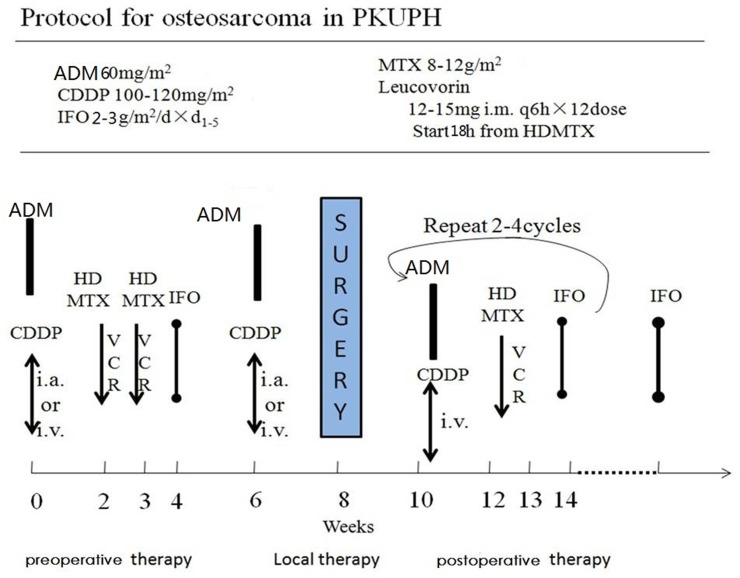
The osteosarcoma chemotherapy protocol for Peking University People’s Hospital

Osteosarcoma is the most common primary bone tumor in the first 3 decades of life and accounts for about 4% of all childhood tumors worldwide [17]. High-dose MTX, adriamycin, cisplatin and ifosfamide have already formed the backbone of most standard treatment protocols of the first-line chemo-regimen [18] which is also the first-line chemo-protocol for Peking University People’s Hospital (Figure 2). Little is known about these chemotherapy pharmacogenomics for osteosarcoma, but information gained from the use of these agents in other malignancies provides contentious discussion about the genetic polymorphisms probably influencing on both chemo-toxicity and outcome in other tumors [1, 19]. Thus this study is aimed to investigate the association of candidate genetic polymorphisms of MTHFR with MTX distribution and metabolism, histological response, survival and grade 3–4 chemotherapy toxicity after treatment with MTX in osteosarcoma.

## RESULTS

### Participants characteristics and genotype information

From January 2012 to May 2015, 62 participants were enrolled; one patient dropped the first-line chemotherapy because of rapidly tumor progression and two patients were lost to follow-up, whom had all been excluded from this study. Clinical and pathological characteristics of study participants are shown in Table 1. All those participants were followed from 29.4 to 69.9 months with median follow-up time of 39.1 months. Among the 59 eligible patients, there were 37 males (62.7%) and 22 females (37.3%). At initial diagnosis, the median age of all eligible patients was 16 years (range, 5 to 52 years) with 6 patients older than 40 yrs. The tumor located at axial skeleton in 12 patients (20.3%), distal femur in 24 patients (40.7%), proximal tibia in 12 patients (20.3%), proximal humerus in 8 patients (13.6%), proximal femur of one patient (1.7%) and distal tibia and fibula in two patients (3.4%).

**Table 1:** Clinical and pathological characteristics of study participants

Characteristics	No. (%)	*P* (Cox univariate and multivariate analysis for overall survival)
Total No. patients	59 (100%)	
MTHFR C677T		*0.777 and 0.849*
CC	15 (25.4%)	
CT	20 (33.9%)	
TT	24 (40.7%)	
MTHFR A1298C		
AA	58 (98.3%)	
AC	1 (1.7%)	
CC	0 (0.0%)	
Age at diagnosis, y		*0.416 and 0.909*
Median (range)	16.0 (5–52)	
Follow-up, mo		
Median (range)	39.1 (29.4–69.6)	
Gender		*0.866 and 0.475*
Male	37 (62.7%)	
Female	22 (37.3%)	
Primary location		*0.089 and 0.056*
axial skeleton	12 (20.3%)	
distal femur	24 (40.7%)	
proximal tibia	12 (20.3%)	
proximal humerus	8 (13.6%)	
proximal femur	1 (1.7%)	
distal tibia and fibula	2 (3.4%)	
Metastasis at diagnosis		*0.004 and 0.033*
Absent	39 (66.1%)	
Present	20 (33.9%)	
Histological subtype		*0.059 and 0.052*
Common type(osteoblastic, chondroblastic, fibroblastic)	36 (61.0%)	
Telangiectatic type	3 (5.1%)	
Histological response		*0.164 and 0.294*
Good (≥90%)	11 (18.6%)	
Poor (<90%)	33 (55.9%)	
Not evaluable	15 (25.4%)	
Death		
Yes	28 (47.5%)	
No	31 (52.5%)	
Progression		
Yes	36 (61.0%)	
No	23 (39.0%)	

Only one patient was tested as heterozygous mutant genotype of A1298C with a mutation frequency of 0.84%, thus no statistical calculation has been done due to its low incidence. As for the incidence of C667T, 15 (25.4%) patients had wild type, 20 (33.9%) had heterozygous mutant genotype and 24 (40.7%) had homozygous mutant genotype with the genic mutation frequency of 57.7%.

All those patients received a total of 398 cycles of HD-MTX, 249 cycles of Adriamycin, 226 cycles of cisplatin and 203 cycles of ifosfamide. The mean initial serum MTX concentration of the 0 hour was 1064.5 μmol/L (95% CI, 995.1–1134.2 μmol/L) (range, 576.2 to 1688.5 μmol/L). While the mean all MTX concentration of the 0 hour was 1070.2 μmol/L (95% CI, 1009.5–1130.9 μmol/L) (range, 621.7 to 1704.0 μmol/L). We adjusted the dose of HD-MTX according to the initial serum MTX concentration for better treatment effect and less drug toxicity according to the guidelines of HD-MTX chemotherapy for osteosarcoma [23–25]. It is understandable that with fluctuating dose of MTX, there is no correlation between C667T genetic polymorphisms and either initial or mean MTX concentration (*P = 0.867* and *0.995*; *r =0.024* and *0.01* respectively) Table 2. There were four times of delayed elimination of MTX with patients’ C677T genotype as TT, CT, CC, CC separately, which also showed no statistical significant difference (*P = 0.305; r = −0.136*).

**Table 2:** Toxicity and oncologic outcomes in relation with genotype frequencies of SNPs in MTHFR C677T

Gene	MTHFR C677T			correlation coefficient (*r*)	significance (*P*)
	CC	CT	TT		
Total cases	15 (25.4%)	20 (33.9%)	24 (40.7%)		
initial serum MTX concentration (0 hr)				*0.024*	*0.867*
mean serum MTX concentration (0 hr)				*0.001*	*0.995*
Delayed elimination of MTX				*−0.136*	*0.305*
Liver toxicity (G3/G4)	2 (3.4%)	11 (18.6%)	19 (32.2%)	*0.482*	*0.043*
Fever after MTX	3 (5.1%)	8 (13.6%)	10 (16.9%)	*0.403*	*0.050*
Myelosuppression III–IV	4 (6.8%)	8 (13.6%)	16 (27.1%)	*0.205*	*0.095*
ulcerous stomatitis	3 (5.1%)	8 (13.6%)	3 (5.1%)	*−0.014*	*0.918*
chronic anemia	3 (5.1%)	1 (1.7%)	3 (5.1%)	*−0.070*	*0.598*
Histological Response (≥90%)	4 (6.8%)	3 (5.1%)	4 (6.8%)	*−0.115*	*0.465*
Overall survival (2yr)	80.0%	55.0%	75.0%		*0.269*
Progression	7 (11.9%)	11 (18.6%)	12 (20.3%)		*0.349*
Local Relapse	5 (8.5%)	6 (10.2%)	3 (5.0%)		*0.146*
Metastasis	7 (11.9%)	11 (18.6%)	12 (20.3%)		*0.371*

### Histological response

On pathologic examination, the surgical specimens were carefully studied and sectioned. This evaluation included establishing the gross extent of the tumor [26, 27] and noting its soft tissue component and lines of surgical resection [27]. An average of 10–20 histologic specimens were examined in each of the en bloc resections to delineate the extension of osteosarcoma up and down the marrow cavity and to study the effects of chemotherapy on the tumor (viable, partially, largely, or totally necrotic), which were then calculated as tumor necrosis rate as paper described [26, 27]. We had done the tumor necrosis rate with all those 59 participants and found that a remarkable relevance with the tumor necrosis rates and the progression-free survival (*P < 0.001; r = 0.527*). However no significant association was observed between tumor necrosis rates and the C677T geno-polymorphism (*P = 0.465; r =-0.107*) Table 2. Data for statistical significance associated polymorphisms are presented in Table 3.

**Table 3:** The study of MTHFR polymorphisms and MTX therapy outcome for osteosarcoma patients

Treatment regimes	Patient population	Race/ethnicity	MTHFR polymorphisms	Main results	Citations
MAP or VAC	Children and adolescents (*n* = 34)	Caucasian origin	C677T, A1298C	Patients with G3/G4 hematologic toxicity were more frequently TT than CT/CC for C677T/MTHFR (*P = 0.023*).	[31]
MAP	Patients aged >16 yrs (*n* = 58)	Caucasian origin	C677T, A1298C, C1305T	Methotrexate toxicity was increased in variants of MTHFR c. 1298A>C (*P = 0.03*).	[11]
APMI or APMIE	Adolescents and adults (*n* = 62)	Caucasian origin	C677T, A1298C	The MTHFR 677C allele was associated with higher degree of liver toxicity (88%, *P = 0.007*).	[30]
APMI	Adolescents and adults (*n* = 59)	Han nationality from mainland of China	C677T, A1298C	Patients with mutation of C677T were associated with higher degree of liver toxicity (*P = 0.043*) and fever reaction of MTX (*P = 0.050*) while G3/G4 hematologic toxicity were more likely to be noticed with TT than CT/CC (*P = 0.095*).	Present study

### Toxicity outcomes

Chemotherapy toxicity was recorded for cycles of chemotherapy. We observed over one third of the patients (*N* = 21, 35.6%) suffered fever two or three days after the infusion of MTX and it was possibly relevant with MTHFR C677T mutant genotype (*P = 0.05; r = 0.403*). Most of the fever was between 37.2 degree centigrade and 38.5 degree centigrade, which should not be dealt with. However three of our 59 patients had fever as high as 39 degree centigrade without obvious manifestation of infection or anaphylaxis, during which we treated them with salicylic acid preparation and cooling therapy. Usually these patients got back to normal 3 to 4 days later spontaneously, which, in our opinions, should also be a phenomenon related with HD-MTX chemotherapy. These patients with genotype CC at MTHFR 677 locus had significantly higher liver toxicity (our continuous variable), measured by CTCAE G3/4 (*P < 0.05; r = 0.482*). Of our patients, nearly 50% (*N* = 28, 47.5%) suffered some kind of hematologic toxicity (neutropenia, leukopenia) to grades G3/G4. And as other researchers have detected in various solid tumors, our analysis also revealed an increased trend for the mutant genotype patients to have G3/G4 hematologic toxicity (*P < 0.05; r = 0.205*). Nevertheless no relativity was found between ulcerous stomatitis (*N* = 14, 23.7%) or chronic anemia (*N* = 16, 27.1%) due to disturbance of folate metabolism and the C677T polymorphisms (*P = 0.918* and *0.598*; *r = −0.014* and *-0.070* respectively) Table 2. Other gastrointestinal toxicity (diarrhea, nausea, vomit and so on) (*N* = 26, 44.1%) and renal toxicity (elevated creatinine, hematuria and proteinuria) (*N* = 2, 3.4%) were also observed frequently in our patients, but they might not be relative with MTX chemotherapy and no relation with genotype mutation was discovered (*P = 0.678* and *0.490*).

### Disease outcomes

**Figure 3:**
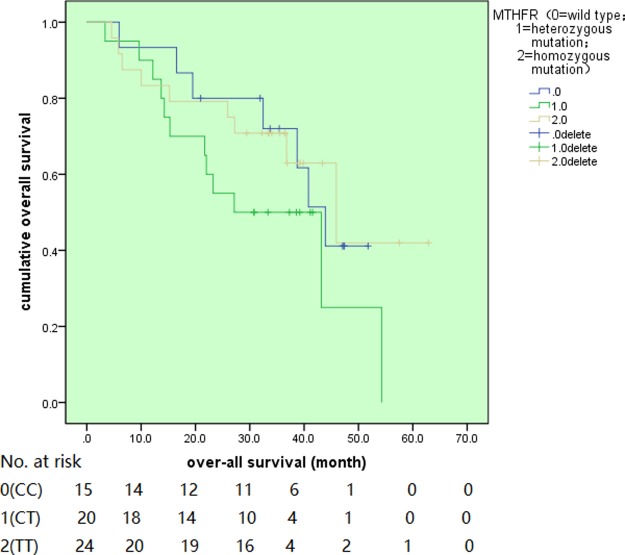
The overall survival for different MTHFR genotypes

**Figure 4:**
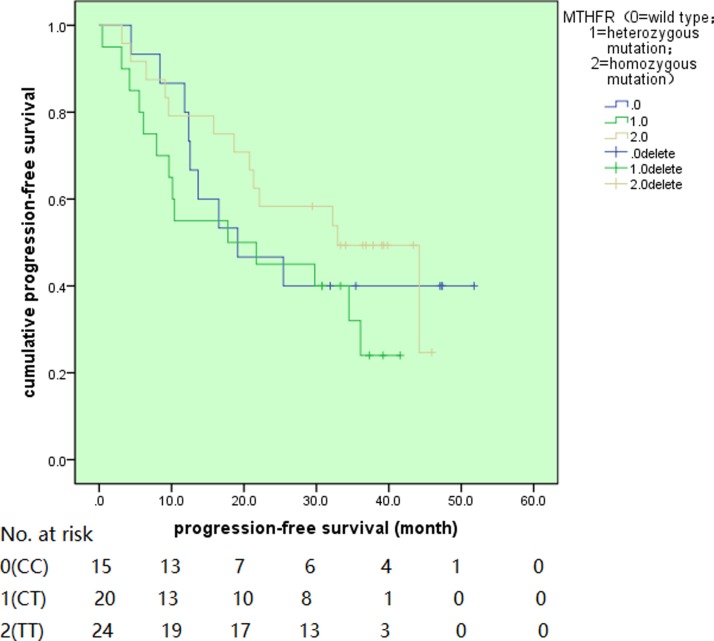
The progression-free survival for different MTHFR genotypes (Transporters: hFR, human folate receptor; RFC, reduced folate carrier. MTX, methotrexate; MTX-PG, methotrexate-polyglutamated; DHF, dihydrofolate; DHFR, dihydrofolate reductase; THF, tetrahydrofolate; MTHFR, 5,10-methylenetetrahydrofolate reductase).

At our last follow-up (median follow-up time of 39.1 month), 28 (47.4 %) patients were dead of the disease, 9 (15.3%) patients were alive with disease and 22 (37.3%) patients were alive with no evidence of disease. For overall survival, patients with wild type of C677T, heterozygous mutant genotype and homozygous mutant genotype had 2-year survival rate of 80.0%, 55.0% and 75.0% respectively without statistical significant discrepancy (*P = 0.269*, shown in Figure 3). At the same time for subgroup analysis, the local recurrence-free survival and metastasis-free survival had both not been found with any relationship with this MTHFR-C677T genetic polymorphisms with *P* as *0.146* and *0.371* separately Figure 4. From the Cox univariate analysis, primary tumor location, whether metastasis at presentation and histological subtypes all had some impact on overall survival (*P = 0.089, 0.004 and 0.059* respectively), the genotypes, gender, age, histological response all had no obvious effect on overall survival (*P = 0.777, 0.866, 0.164* respectively). As for Cox multivariate analysis, these three factors still had slight impact on overall survival with *P* value as *0.056, 0.033* and *0.052* respectively.

## DISCUSSION

In our study most people were from the north of Chinese mainland with no 1298CC mutant genotype but only one patient with 1298 allele (1.7%), which seemed to be a little bit lower than other research [1] and thus this data could not proceed to do the statistical calculations with A1298C. However our other genotype MTHFR C677T has 33.9% 677 allele and 40.7% 677TT, which was in accordance with the north of China mainland data [28]. Those demographic incidence differences may due to the small size of our sample. For histologic response and overall survival, patients with wild type of C677T, heterozygous mutation and homozygous mutation did not have obvious different distribution of tumor necrosis rate (*P = 0.465* and *r = −0.115*) and they had 2-year survival rate of 80.0%, 55.0% and 75.0% respectively without statistical significant discrepancy (*P = 0.269*). As for toxicity, patients with genotype TT at MTHFR 677 locus had significantly higher liver toxicity (*P < 0.05; r = 0.482*). Our analysis also revealed an increased trend for the mutant genotype patients to have G3/G4 hematologic toxicity (*P < 0.05; r = 0.205*). Nevertheless no relativity was found between ulcerous stomatitis (*N* = 14, 23.7%) or chronic anemia (*N* = 16, 27.1%) due to disturbance of folate metabolism and the C677T polymorphisms (*P = 0.918 and 0.598; r = −0.014 and −0.070* respectively).

Some of the literatures reported these MTHFR gene polymorphisms had some impact on survival of pediatric acute lymphoblastic leukemia, while most of the articles stated that they had no effect on recurrence-free survival or event-free survival [19, 29]. Nearly half literatures reported a significant correlation between the C677T variant genotype and an increased risk of developing adverse events (grades 3 and 4 hematologic and non-hematologic toxicity) after treatment with MTX alone or in combination with other agents, in patients with hematologic malignancies [30], ovarian [31] and breast cancers [32] and even patients with rheumatoid arthritis or juvenile idiopathic arthritis [33]. While other reports shared opposite results [14, 34–36]. There are few reports discussing MTHFR polymorphisms in osteosarcoma [36] (summarized in Table 3). From 2009 to 2015, three literatures [14, 35, 36] investigated the potential effects of MTHFR on osteosarcoma patients and were all focused on Caucasian origin. It seemed this MTHFR indeed had some impact on the toxicity of MTX but not on the disease outcomes and histological responses. We explored the detailed MTX plasma concentration and metabolism of osteosarcoma in mainland of China all with Han nationality. The purpose of this study is to explore the effect of MTHFR polymorphisms on MTX chemo-toxicity and oncologic outcomes of particularly Chinese osteosarcoma patients.

At our last follow-up (median follow-up time of 39.1 month), 28 (47.4 %) patients were dead of the disease, 9 (15.3%) patients were alive with disease and 22 (37.3%) patients were alive with no evidence of disease. This high incidence of fatality may due to the high rates of axial skeleton osteosarcoma, which was reported to have much more poorer prognosis than that of extremities [37]. In our series of pediatric, adolescent and adult osteosarcoma, none polymorphic variants was significantly related to prognosis (overall survival, progression-free survival, local relapse-free survival and development of metastasis) or to the response to treatment (tumor necrosis induced by the neo-adjuvant chemotherapy). This may due to the multiple drugs’ combination therapy. And the situation was almost the same in other solid tumors [19]. For breast cancer, there were 17 significant investigations and 17 non-significant investigations at MTHFR gene C677T on toxicity [38–42]. While there was only one study presented with different oncologic prognosis [42]. From the available data, the most evident association emerging is between the C677T variant form and increased MTX toxicity [29, 32, 36, 41]. Converse correlations between C677T and MTX efficacy presented a pool of heterogeneous and sometimes contrasting results that provided equivocal conclusions [1, 19, 36, 43].

Patients with genotype TT at MTHFR 677 locus had significantly higher liver toxicity (*P < 0.05; r = 0.482*), mainly measured by ALAT scores [36]. This was in accordance with the study of S Jabeen *et al.* [36] They investigated 62 osteosarcoma patients and found out that MTHFR C677T with mutant genotypes had higher rate of ALAT score. But the mucositis score was nearly the same among different genotype groups, which was also like the conclusion our study have reached with *P = 0.918* and *r = −0.014*. But another point, which should be revealed in our study, was that our patients mostly received leucovorin calcium medical mouthwash to gargle in order to reduce the mucosal erosion of the mouth, thus our patients’ not obvious difference may partly due to the mouthwash, so this comparison may be meaningless.

We observed over one third of the patients (*N* = 21, 35.6%) suffered fever usually two or three days after the dripping of MTX and it was possibly relevant with MTHFR C677T mutant genotype (*P = 0.05; r = 0.403*). In a study from Japanese scholar Kagawa Y *et al.* [44], the development of fever is one of the main risk factors for the delayed elimination of MTX. However in our study different C677T genotypes all had delayed elimination of MTX, which had turned out not to be statistical significant (*P = 0.305; r = −0.136*). But still we could observe some individual discrepancy among those patients with different responses to the MTX therapy. Maybe later with more large population scale we can find out some clues.

As other researchers have detected in various solid tumors, we found some trend of a correlation between the MTHFR C677T allele and hematologic toxicity (neutropenia, leukopenia to grades G3/G4). However as for chronic anemia due to disturbance of folate metabolism, there seemed not to be significant (*P = 0.598*; *r = −0.070*). It has also been consistently demonstrated that physiopathological consequences of MTHFR genetic variants, especially the C677T polymorphism, are significantly affected by environmental factors such as folate status, age, smoking and alcohol intake, all parameters that may additionally alter the final equilibrium of one-carbon metabolism [45, 46].

This exploratory study has demonstrated the MTHFR polymorphisms might influence the drug toxicity of MTX and it is the first article investigating the detailed MTX plasma concentration and metabolism of osteosarcoma in mainland of China all with Han nationality. Nonetheless the total number of the current total population is not large enough to discriminate the effect of this MTHFR polymorphisms and the relative low median age of 16 years made this group of people might not be so sensitive to this geno-polymorphisms. Thus this may deserve validation in a larger, prospective cohort.

High dose MTX (HD-MTX) shows continued effectiveness in the treatment of childhood and adult osteosarcoma [47]. Because of an absent transport binding mechanism, osteosarcoma is resistant to conventional doses of MTX [48]. However it may still be susceptible to high doses of MTX. The exact mechanism of this action is not fully established, but is postulated to be related to the transport mechanism that methotrexate enters the osteosarcoma cell by passive diffusion, which needs extracellular concentrations attained with the massive doses over time to force it entering into cells [47]. As one of the first agents that held promise to the prognosis of osteosarcoma, HD-MTX has never wavered its status in the chemotherapy treatment of osteosarcoma since mid-1970s [49, 50]. Methotrexate with leucovorin rescue is optimally administered as 10–12.5 g/m^2^ over 4–6 hours in 600 cm^3^ of fluid followed by leucovorin rescue 10–12 mg as a loading dose at 24 hours after commencement of therapy, which was maintained at the same dose at 6 hour intervals. It may be discontinued when a serum methotrexate level of 0.1 mol/L is obtained [47]. Intravenous fluids should be maintained at 3 L/m^2^/24 h. In some centers, the dose of leucovorin is calculated according to the weight or body surface area. Not everybody could endure this HD-MTX chemotherapy. There were lots of literatures and trials [8, 10–12, 14, 35, 36, 51] discussing about whether it was appropriate for older people (mostly age older than 40 years) to receive HD-MTX mainly because of a serious complication named as “delayed elimination” of HD-MTX, which was also one of the main reasons that older osteosarcoma patients had poorer prognosis than younger patients [52].

According to Zelcer *et al.* [23], 24 hours post-administration, 78% of the MTX levels were ≤20 μmol/L. By 72 hours post-MTX, the majority of MTX levels (62%) were ≤0.1 μmol/L, a level at which the drug was considered adequately cleared. A minority of levels were tested as >20 μmol/L at 24 hours, or >1–2 μmol/L at 48 hours or even >0.1–0.2 μmol/L at 72 hours post MTX infusion [20, 21, 23], a concentration at which the risk of clinical toxicity is believed to be high and admission is mandatory according to MSKCC guidelines [23]. This is usually called “Delayed MTX elimination”, which was believed to be the most serious complications after HD-MTX and caused lots of patients died in the past time. It is reported that this phenomenon is associated closely with liver toxicity and severe gastrointestinal mucositis [21], which would cause the patient unable to eat and further lack of raw material for hematopoiesis. Usually the patient would undergo approximately 3–7 days’ period of IV grade myelo-suppression after the MTX therapy. Some literature [22] pointed out that patients with the C677T genotype present a significant higher serum concentration of MTX 48h after initiating the therapy compared with patients with other genotypes. However in our study, there were only four cases of delayed elimination of MTX present, of which patients’ C677T genotype were as TT, CT, CC, CC separately, which also showed no statistical significant difference (*P = 0.305; r = −0.136*).

## MATERIALS AND METHODS

### Participants and samples

From January 1st 2012 to May 1st 2015, patients who met the following criteria were included: 1) histologically confirmed high-grade osteosarcoma; 2) initially treated in Musculoskeletal Tumor Center of Peking University People’s Hospital; 3) Serum samples were available; 4) completed neo-adjuvant chemotherapy and at least 8 cycles of adjuvant chemotherapy (Figure 2); 6) expected to live longer than 3 months with Eastern Cooperative Oncology Group performance status of 0 or 1 and acceptable hematologic, hepatic, and renal function. Patients who have not completed neo-adjuvant chemotherapy or at least 4-month adjuvant chemotherapy were excluded from this study. Finally serum samples were available for 62 HD-MTX-treated osteosarcoma patients, which had all been taken before the first chemotherapy.

All the patients in this study followed the chemo-protocol as Figure 2 with different time-intervals for adjuvant chemotherapy because of chemo-tolerance. The study protocol was approved by the Ethics Committee of Peking University People’s Hospital. Written informed consent was obtained from all participants.

Two 5 ml ethylenediaminetetraacetic acid venous blood samples were obtained from all subjects at recruitment. MTHFR mutant genotype was detected by standard polymerase chain reaction(PCR) and PCR-restriction fragment length polymorphism techniques were described previously in Hardy-Weinberg equilibrium [53].

Generally speaking patients were treated before surgery with intravenous cisplatin of 100–120 mg/m^2^ at day 1 followed by doxorubicin (2 courses at 20 mg/m^2^/d for 3 days), intravenous double MTX (10–12 g/m^2^ once a week twice) and at last followed by ifosfamide (2–3 g/m^2^/d for 5 days). And after surgery the adjuvant chemotherapy was followed by 12–16 cycles depending on the patient’s personal constitution and tolerance (Figure 2).

MTX concentration was measured in 4 serum samples (O hour, 24 hours, 48 hours and 72 hours at the beginning of the infusion) per HD-MTX cycle in all 59 consecutive patients. Alanine aminotransferase (ALAT), glutamic oxaloacetic aminotransferase (ASAT), alkaline phosphatase, gammaglutamyltransferase and total bilirubin, direct bilirubin were measured in the same serum samples by methods in routine clinical practice, which had been described by Holmboes *et al.* [54]. Hematologic, gastrointestinal and renal toxicities were recorded and graded according to Common Terminology Criteria for Adverse Events version 3.0 (CTCAE) [55].

### Statistical analysis

The genotype frequencies of SNPs with minor allele frequency <0.05 were excluded. Pearson chi-square or Fisher exact tests were used to analyze clinical, pathologic and genotypic data. The CTCAE grades were grouped as binary variables (grade 0–2; grade 3–4) for each drug cycle. Associations between genotypes and toxicity were calculated by double correlation analysis and presented as correlation coefficient (*r*) and their significance (*P*).

The primary objective was to describe the relationship between MTHFR polymorphism and the efficiency and toxicity of MTX. Endpoints were overall survival (OS) and the characterization of toxicities. The secondary objective was progression-free survival (PFS) and histological response. Disease recurrence was defined as tumor progression including recurrence with pulmonary metastasis or bone metastasis and local relapse. Progression-free survival (PFS) was calculated from the start of chemotherapy to first disease recurrence. Patients without disease recurrence at final analysis were censored at last follow-up. Overall survival was calculated from the start of chemotherapy to death. The Kaplan-Meier method was used to compare differences in PFS and OS. Cox proportional hazards model was used for calculation of hazard radio (HRs) in multivariate analysis as covariates. All statistical analyses were performed using SPSS version 19.0 (SPSS Inc., Chicago, III). Reported *P* values were two-sided and a value of *0.05* was considered statistically significant, while a value of *0.1* was supposed as slight trend. Adjustment for multiple testing was not performed, as *P* values and CIs were felt to be more informative in the setting of a small pilot study.

## CONCLUSIONS

This study suggested that genetic polymorphism of MTHFR C677T might not help to find delayed elimination of MTX but might have some relationship with liver toxicity and fever after MTX therapy. Other toxic and side effects were not obvious among different genetic groups. MTHFR genetic polymorphisms have no obvious effect on histologic response or survival in osteosarcoma patients.
